# The National Health Summit: Bridging Policy and Practice for Transformative Healthcare in Nepal

**DOI:** 10.31729/jnma.8872

**Published:** 2025-01-31

**Authors:** Sushant Regmi

**Affiliations:** 1 Managing Editor, Journal of Nepal Medical Association, Exhibition Road, Kathmandu, Nepal

## INTRODUCTION

The National Health Summit (NHS) is a calendar event organized by the Nepal Medical Association (NMA) with Journal of Nepal medical Association (JNMA) as the scientific lead. NHS serves as a platform that aims to bridge the gap between the evidence based-reasearch and implementing policies into action.

NHS was initiated with a vision to address the gap between the research findings and their implementation in health policy. Prior summits that were conducted in Nepal often focused on research pertaining to disease. So NHS was designed to serve as a think tank for health policy innovation.

The NHS aligns closely with the National Health Policy 2019 (NHP-2019), which emphasizes universal health coverage, equitable access to healthcare, and multisectoral collaboration for health development.^1^ Similarly, the Nepal Health Sector Strategic Plan 20232030 (NHSSP 2023-2030) focuses on strengthening the healthcare system, addressing disparities in healthcare delivery, and fostering innovation to improve health outcomes.^2^ These guiding frameworks shape the summit's agenda, ensuring its relevance and alignment with national health priorities.

The first health summit, held during the COVID-19 pandemic, laid the foundation for long-term policy discussions. Initial summits was limited to policy makers and public health professional and the most recent summit has evolved into a multidisciplinary forum.

Now in its fourth year, the NHS emphasizes broad policy-level discussions under the theme "Health Beyond Health". Health Beyond Health,' emphasizes actionable, multidisciplinary solutions to pressing national health concernsThe NHS serves as a venue for high-level panel discussions involving policymakers, healthcare professionals, and other stakeholders. While the summit does not focus on research presentations, it provides an opportunity for dialouge on critical health issues and strategies through a panel discussion. The panel discussions are based on sub themes supporting the main theme of the summit. From this year onward, the JNMA team has initiated a policy research project to make NHS more scientific and evidence-based. This initiative reflects JNMA's dedication to grounding healthcare advancements in data and analysis.

## THE ROLE OF THE JOURNAL OF NEPAL MEDICAL ASSOCIATION (JNMA)

The NHS, organized by the NMA with support from JNMA, has made significant progresses in driving policy-oriented discussions. The inaugural and second summits, conducted in September, align with the founding month of the JNMA, highlighting the journal's commitment to linking research with policy.

From this year onward, the JNMA has initiated a policy research project to make the NHS more evidence-driven. The ongoing policy research effort aims to strengthen NHS as a platform for shaping Nepal's healthcare outlook, ensuring its sustainability and lasting impact This effort reflects the JNMA's dedication to grounding healthcare reforms in data and analysis, ensuring that the summit's outcomes are actionable and impactful.

## IMPACT OF SUMMIT

Through its sessions, the NHS has facilitated discussions on critical topics such as universal health coverage, maternal and child health, and pandemic preparedness. The summit ensures its relevance by addressing both global health priorities and Nepal's specific healthcare challenges.

Globally, health summits like the World Health Assembly (WHA) have demonstrated the transformative power of multilateral collaboration, influencing international health policies and programs.^3^ Drawing inspiration from such assemblies, the NHS has prioritized inclusivity and evidence-based decision-making, positioning itself as a model for national health governance.

## CONCLUSION

The summit's evolution highlights its emphasis on diverse stakeholder discussions and sustainable, evidence-based health governance. By addressing current challenges, the NHS has the potential to drive transformative change in Nepal's healthcare system.
